# A Multilevel Model of Entrepreneurship Education and Entrepreneurial Intention: Opportunity Recognition as a Mediator and Entrepreneurial Learning as a Moderator

**DOI:** 10.3389/fpsyg.2022.837388

**Published:** 2022-02-11

**Authors:** Fei Hou, Yu Su, Mingde Qi, Jun Chen, Jiayun Tang

**Affiliations:** ^1^Institute of Advanced Studies in Humanities and Social Sciences, Beijing Normal University, Zhuhai, China; ^2^Management School, Guangdong University of Technology, Guangzhou, China; ^3^Management School, Guangdong Peizheng College, Guangzhou, China

**Keywords:** entrepreneurship education, opportunity recognition, entrepreneurial learning, entrepreneurial intention, multilevel-moderated mediation model

## Abstract

Highlighting the implications of entrepreneurship education, this study examines the effects of entrepreneurship education in predicting the entrepreneurial intention of university students. The study also explores the mediating role of opportunity recognition and the moderating role of entrepreneurial learning in this process. To test our multilevel-moderated mediation model, based on a dataset containing 1,150 university students from 55 universities in the Guangdong-Hong Kong-Macao Greater Bay Area of China, hierarchical linear modeling is utilized to test the research hypotheses. The findings reveal that entrepreneurship education can promote the entrepreneurial intention of students through opportunity recognition. Furthermore, entrepreneurial learning plays a moderating role in the link between entrepreneurship education and opportunity recognition. Implications for the design and delivery of entrepreneurship education are discussed.

## Introduction

Given that entrepreneurship acts as a key catalyst in enhancing innovation and competitiveness (Alrawadieh et al., 2019; Booth et al., 2020), it has increasingly become a significant topic among researchers and practitioners worldwide (Fayolle and Gailly, 2008). Shane and Venkataraman (2000) noted that entrepreneurship is an intentional and planned behavior that could promote economic efficiency, introduce innovation, create new jobs, and increase the level of employment. Most empirical evidence that has been provided demonstrates that entrepreneurship, or at least some aspects of entrepreneurship, can be taught, and education has been regarded as instrumental in fostering and enhancing entrepreneurial attitudes, intentions, competences, and ways of thinking and analyzing the world (Falkäng and Alberti, 2000; Harris and Gibson, 2008; Neck and Greene, 2011; Fayolle, 2013; Martin et al., 2013). Indeed, entrepreneurship education (EE) conceptualizes a system of university education that lays a foundation for managing competing current and future industrial demands of innovation and opportunity exploration, and it has offered the most explicit guidance on ways to train students in entrepreneurship (Horng et al., 2020). Hence, EE has increasingly expanded, and the associated benefits have been highlighted by researchers and educators. Accordingly, a dramatic growth in entrepreneurship education (EE) in universities worldwide has been triggered by these studies (Finkle and Deeds, 2001; Katz, 2003; Kuratko, 2005; Matlay, 2005), and investment in EE-related programs is still on the rise.

Nonetheless, extant research has largely failed to assess the outcomes and effectiveness of EE programs (Pittaway and Cope, 2007; von Graevenitz et al., 2010). An important goal for further research should be to evaluate the effectiveness of EE programs (Karimi et al., 2016). Accordingly, an important research question arises: How should EE be evaluated? Considering that the target behavior, which involves unpredictable time lags, is rare and difficult to observe, social psychologists have noted that intentions have been the best predictor of planned behaviors (Krueger et al., 2000). One typical example of these planned and intentional behaviors is entrepreneurship (Bird, 1988; Krueger and Brazeal, 1994). According to Bird (1988), EI is defined as a state of mind that is capable of guiding and directing individual actions toward the development and implementation of a new venture concept. A vast body of research has noted that EI could play a significant role in the decision of whether to start a new venture (Liñán and Chen, 2009). Given that the preference for self-employment is an important indicator of actual involvement in new venture creation (Verheul et al., 2012; Kautonen et al., 2015; Bonesso et al., 2018), EI has also been identified to serve as a key catalyst behind the introduction of new technologies in associating existing resources with innovative concepts to develop and commercialize new products or services, triggering business entry into niche markets and obtain a competitive advantage (Alrawadieh et al., 2019; Horng et al., 2020). In addition to this finding, entrepreneurship researchers have recently developed considerable interest in employment status choice models focusing on EI (e.g., Engle et al., 2010; Iakovleva et al., 2011; Karimi et al., 2014). Indeed, intention models provide a great opportunity for enhancing the sound understanding and predictability of entrepreneurship (Krueger et al., 2000).

Hence, evaluation of individuals’ intentions to establish a new venture is one of the most common ways of assessing EE programs. Intentionality directed toward entrepreneurship is central to the process of entrepreneurial activities (Bird, 1988; Krueger, 1993), and EI is a key antecedent of entrepreneurial behavior. Indeed, the effect of EE programs on EI has still been underexplored (Peterman and Kennedy, 2003; Bechard and Gregoire, 2005; Pittaway and Cope, 2007; Athayde, 2009; Karimi et al., 2016; Gielnik et al., 2017) and has hitherto been poorly tested (Souitaris et al., 2007; von Graevenitz et al., 2010). Furthermore, although the benefits of EE have been established, prior studies have had mixed results. Most of these studies demonstrated that EE programs have a positive influence (e.g., Peterman and Kennedy, 2003; Fayolle et al., 2006; Souitaris et al., 2007; Athayde, 2009; Walter et al., 2013; Kautonen et al., 2015; Westhead and Solesvik, 2016). However, on some occasions, other studies have provided evidence that shows an insignificant or even negative impact of EE programs (e.g., Mentoor and Friedrich, 2007; Oosterbeek et al., 2010; von Graevenitz et al., 2010). Considering that findings concerning EE initiatives remain somewhat inconsistent, these inconclusive results indicate that the effects of EE should be further explored from different perspectives to obtain a sound understanding of the relationship between EE and EI.

Previous studies examining the relationship between EE and EI have mostly regarded EE as an individual-level factor (e.g., students’ perceived EE; Walter and Block, 2016). In this regard, other studies have investigated the organizational-level factors influencing entrepreneurial interest and behavior, such as organizational norms (Louis et al., 1989), university quality (Di Gregorio and Shane, 2003), and EE (Souitaris et al., 2007; Walter et al., 2013). In light of the fact that scholars focused on organizational behavior have claimed that “ultimately, behavior is determined by both dispositions and situations” (House et al., 1996), this study attempts to explore how EE influences EI by considering EE as an organizational-level factor (situational variable), which has been omitted by extant empirical research.

Effective opportunity recognition as a result of increasing entrepreneurial awareness is the first stage and a key catalyst of the entrepreneurial process (Baron, 2006). Through EE programs, entrepreneurs might enhance greater sensitivity to the market and identify changes in the technological environment, thus resulting in learning how to recognize and pursue new business opportunities (Lee et al., 2016). The EE literature has indicated that EE programs (e.g., case studies, contact with entrepreneurs, and entrepreneurial-related activities) provide domain-specific experiences that play a crucial role in facilitating entrepreneurial awareness and ways of thinking (Politis, 2005; Krueger, 2007). These EE programs that are concerned with entrepreneurial cognition and experiential learning could foster participants in developing an entrepreneurial way of observing the world and thus endow them with the capability to recognize business opportunities effectively (Baron, 2006; Anderson and Jack, 2008; Bae et al., 2014). However, EE’s impacts through OR-based indicators have been underexplored by the current literature. As such, this study attempts to fill this gap by examining EE program-derived benefits from an OR perspective and further exploring the OR-based mediating mechanism for the way in which EE programs facilitate the EI of university students.

Equally important is EL, which refers to the process through which newly formed knowledge with preexisting structures is acquired, assimilated, and organized (e.g., Minniti and Bygrave, 2001; Rae and Carswell, 2001; Cope, 2005; Harrison and Leitch, 2005; Corbett, 2007). Specifically, EL has two distinct theoretical frameworks: experiential learning and vicarious learning. Experiential learning refers to the notion that new knowledge is assimilated through the transformation of experience. Conversely, vicarious learning, also called observational learning, is defined as modeling the behaviors of others. Illustrating the substantial importance of this notion, it is imperative to gain a better understanding of EL due to the fact that entrepreneurs develop and grow through learning (Cope, 2005). In view of the EL literature, the effectiveness of prior knowledge and learning processes on newly accumulated knowledge has been explored, and the ways in which the accumulation of new knowledge influences action have been examined. A previous study theoretically explored the impact of EL on OR, indicating that EL is a key predictor for OR (Politis, 2005). However, the primary question that seems to remain unanswered in the extant literature is the question of the role that EL plays in the relationship between EE programs and OR. Hence, this study examines the role of EL as a boundary condition that distinguishes whether deviations in perceived EE are positively or negatively associated with OR and subsequently EI.

These inconsistent results might also be attributed to methodological limitations (von Graevenitz et al., 2010). For example, prior studies have mostly been ex ante and ex post-surveys that fail to evaluate the direct impact of EE programs (e.g., Kolvereid and Moen, 1997; Menzies and Paradi, 2003), and these studies have generally had smaller sample sizes (e.g., Fayolle et al., 2006; Jones et al., 2008). Along with the arguments discussed thus far, most prior studies have focused on investigating how individual-level EE has an effect on subsequent EI. By considering university-based EE as a situational variable, this study attempts to perform a cross-level analysis concerning how organizational-level EE affects student and graduate entrepreneurship. Based on a dataset containing 1,150 university students from 55 universities in the Guangdong-Hong Kong-Macao Greater Bay Area (GBA) of China, hierarchical linear modeling (HLM) is utilized to test the research hypotheses.

**Figure 1:**
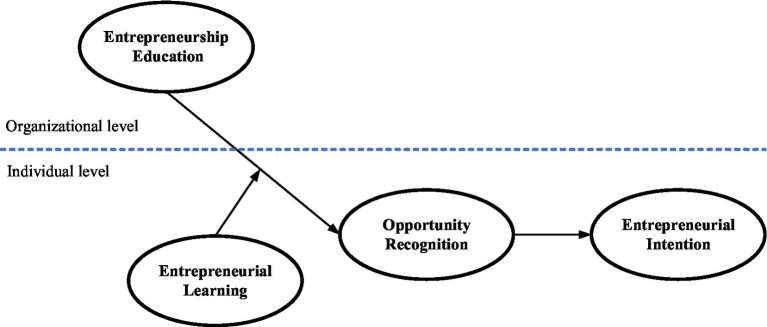
Conceptual model.

To reduce these theoretical and methodological gaps, this study contributes to the EE literature on multiple fronts. First, this study extends the research concerning the effects of individual-level EE to an investigation of the impacts of organizational-level EE *via* multilevel analysis. Second, on the basis of an OR-based perspective, this study contributes novel ways of assessing university-based EE program-derived benefits and identifies the underlying mechanism to uncover how EE fosters future entrepreneurs. Third, this study also extends prior research concerning EL by exploring its function as a boundary condition that could explain why university students differ in their levels of sensitivity to the impacts of EE programs with regard to OR and EI. Our study provides some of the first empirical evidence to examine how EE, as the organizational context, facilitates shaping future entrepreneurs from an OR-based perspective. This insight also aids EE policy-makers and educators in justifying investments in and optimizing designs for EE programs. The conceptual model of this study is presented in Figure 1.

## Theoretical Orientations

### Entrepreneurship Education as an Organizational-Level Factor

In exploring why individuals identify, assess, and exploit opportunities in the context of entrepreneurship, there are two streams of arguments. One type is an individual-level argument that refers to individual factors, including achievement orientation, risk tolerance, and independence seeking behavior. In contrast, the other type is the organizational-level argument, which pertains to organizational characteristics, including university policies, characteristics of the technology licensing office, university culture, and intellectual eminence (Shane, 2004). Notably, studies have provided empirical evidence that university-based EE programs have a positive influence on the EI of students (Peterman and Kennedy, 2003; Souitaris et al., 2007). In this regard, a cross-level analysis could help to gain a better understanding of the context in which individual behavior takes place and how that context exerts cross-level influence on within-group level behavior. As such, the cross-level approach is conducive to explaining the controversy concerning the mixed results of prior studies focusing on individual-level factors (Walter et al., 2013).

In light of the study by Hoegl et al. (2003), critical characteristics at the team level might exert more influence on “insiders” than on “outsiders.” In this vein, considering when team members are assimilated in the process of socialization by team-level properties, contextual factors function regardless of individual preferences. According to Walter et al. (2013), based on the contextual effect approach, “pro-entrepreneurship” universities foster stronger norms that are valuable for entrepreneurship and are more likely to promote student self-employment. Given this reasoning, EE can be considered an organizational-level characteristic that fosters a contextual norm, influencing students’ attitudes and intentions toward entrepreneurship.

In contrast to previous studies on student entrepreneurship that have been conducted on the basis of pre- and post-test quasi-experimental designs, a cross-level lens can complement and extend this field to accurately evaluate the effectiveness of EE programs, as organizational characteristics, on students’ perceptions and attitudes toward entrepreneurship. Along with these insights, this study attempts to investigate how EE programs act as contextual effects to affect the EI of university students by adopting a cross-level approach and then to fully understand the mechanism underlying this cross-level relationship.

### Entrepreneurship Education and Entrepreneurial Intention

EE refers to the scope of curricular courses or programs that specifically focus on sensitizing and qualifying participants for entrepreneurship-related activities. As seen from the literature review, EE programs can generate various entrepreneurial outcomes (Pittaway and Cope, 2007; Mojab et al., 2011; Sánchez, 2013); however, there is still debate regarding whether entrepreneurship can be taught (Aronsson, 2004; Gendron, 2004; Walter et al., 2013).

Education and training could foster entrepreneurship-related elements, resulting in transferring human capital associated with entrepreneurship and enhancing business opportunity recognition (Henry et al., 2005; Ucbasaran et al., 2008). In this regard, the methods and techniques, including business planning and market analysis, offered by EE programs and training could aid in recognizing valuable business ideas. Furthermore, by transferring entrepreneurship-specific human capital, EE programs and training can enhance identification and exploitation of business opportunities, resulting in potential yields. Hence, some key entrepreneurship-related elements have been explored, including the transition from business ideas to the market, strategies for market entry, resource acquisition, and ways of organizing and managing a new business.

In the study by Souitaris et al. (2007), university students majoring in science and engineering were adopted as the research sample to empirically test the relationship between EE programs and the EI of students. Their results showed that EE programs could significantly enhance the attitudes and intentions of university students. Peterman and Kennedy (2003) and Athayde (2009) used high school students as samples to test the impact of EE programs on intentions, and a positive result was obtained. In particular, according to the meta-analysis by Martin et al. (2013), overall positive impacts of EE programs on knowledge and skill, perceptions toward entrepreneurship, and entrepreneurship-related outcomes have been found.

Notably, according to Souitaris et al. (2007), EE programs refer to a portfolio of activities regarding entrepreneurship, which is broader than one specific course. In the analytical framework of their study, balanced EE programs that exhibited “good practice” were analyzed in terms of four components: (1) the “taught” component: entrepreneurial courses and lectures; (2) the “business-planning” component: business idea development and business plan competition; (3) the “interaction with practice” component: interacting with real entrepreneurs and networking events; and (4) the “university support” component: market-research resources, space for meetings, a pool of technology with commercial potential, and even seed funding for student teams.

In this vein, drawing on the perspective of organizational norms, pro-entrepreneurship norms could be fostered by EE programs, which would render to the EI of participants socially desirable. Indeed, evidence suggesting a positive link between perceived social desirability and EI has been robust (Kolvereid, 1996; Peterman and Kennedy, 2003). As such, entrepreneurship-specific organizational norms could strengthen members’ perception of entrepreneurship as a legitimate alternative career choice and enhance their self-employment preferences.

Thus, drawing on the above discussions concerning the effects of EE on EI, this study proposes the following hypothesis:

*Hypothesis* 1: Entrepreneurship education is positively related to entrepreneurial intention.

### The Mediated Effect of Opportunity Recognition

According to Lumpkin and Lichtenstein (2005), OR refers to the capability to identify a good idea and transform it into a business concept that could enhance value to the customer or society as well as yield revenue for the entrepreneur. Considering that OR has long been considered a key component in the process of entrepreneurship (Shane and Venkataraman, 2000; Gaglio and Katz, 2001; Ardichvili et al., 2003; Ozgen and Baron, 2007), entrepreneurship cannot take place without the identification of business opportunities (Short et al., 2010). Hence, in academic research and studies of entrepreneurship, OR has been regarded as a prerequisite, and scholars have developed an intense interest in exploring the antecedent factors and dynamic processes that could foster it (Grégoire et al., 2010). In this respect, there are two main theories concerning OR: the discovery theory and the creation theory (Alvarez and Barney, 2007). Recently, research evidence has been provided to support the claim that both main approaches could take place in the practice of entrepreneurship (Short et al., 2010).

According to Dutta et al. (2011), the transformation from potential entrepreneurs to real entrepreneurs depends on the creation and management of new businesses. In this regard, not only the task of fostering an intention toward entrepreneurship but also the identification and exploitation of business opportunities in a timely and effective fashion are essential for this transformation. As such, considering that opportunity identification plays a pivotal role in the process of entrepreneurship, EE programs should promote opportunity identification competency (Liñán et al., 2011). A review of recent literature concerning EE shows that OR can be taught and that EE programs should place OR in a central position to foster and transform potential entrepreneurs (Saks and Gaglio, 2002).

In light of the EE literature and research (Politis, 2005; Krueger, 2007), entrepreneurial awareness of participants could be fostered and developed by specific field experiences resulting from EE programs or training, such as case studies, contact and interaction with entrepreneurs, and engagement in entrepreneurial activities. In this respect, the cognitive perspective has been regarded as crucial to the development of EE due to the fact that entrepreneurial principles are not based on inheritance or stability (Palich and Bagby, 1995; Baron, 2006). In contrast to trait theory, which emphasizes personality traits that are stable and inherited, and that endure over time (e.g., Palich and Bagby, 1995; Cope, 2005), cognitive theory notes that individuals can foster and enhance their cognitive frameworks through significant experiences, which are transformed into knowledge. According to Corbett (2007), the transformation of significant experiences into knowledge represents a process of learning that is contextualized within a given situation. Hence, because cognitive frameworks can orient individuals to be sensitive to contextually specific information pertaining to effective OR, these frameworks have been regarded as crucial to training potential entrepreneurs to foster entrepreneurial awareness and mindset (Baron, 2006; Krueger, 2007). In this sense, potential entrepreneurs are able to learn how to recognize opportunities through developing the cognitive frameworks of OR and through developing competencies regarding entrepreneurship based on EE (Morris et al., 2013).

In line with this reasoning, EE-related courses and training lay the foundation for enhancing individuals’ skills and abilities, which is a prerequisite for opportunity identification (DeTienne and Chandler, 2004). Although a majority of the literature has focused on OR and its role in the process of entrepreneurship, there is only a small body of research concerning the impacts of EE programs on participants’ competence to recognize opportunities. Indeed, there is empirical evidence to support the claim that EE programs can foster participants’ competence to identify opportunities, resulting in more business and innovative opportunities being recognized (DeTienne and Chandler, 2004; Munoz et al., 2011). In light of this reasoning, given that the entrepreneurial knowledge of participants could be amplified by EE programs (Martin et al., 2013), the link between entrepreneurial knowledge and recognition of business opportunities is positive (Shepherd and DeTienne, 2005). Hence, this study suspects that university students who have engaged in EE programs will have higher levels of OR.

There are two events closely related to entrepreneurship: the presence of a suitable opportunity for entrepreneurship and an individual who has the willingness and capabilities to take advantage of an opportunity for entrepreneurship. Indeed, the simultaneous occurrence of two events lays the foundation for the actions required for entrepreneurship and the creation of a new venture (Krueger and Brazeal, 1994; Karimi et al., 2016). Entrepreneurial behavior might be triggered and a new venture created given the coincidence of these two events. A potential entrepreneur has been defined as an individual who perceives an entrepreneurial opportunity and intends to establish a new business firm but who has not yet taken any actions to start a venture (Karimi et al., 2016). Based on these facts, OR and EI are crucial characteristics of potential entrepreneurs, and both simultaneous occurrences warrant the creation of new ventures.

Given that cognitive-related factors could play a crucial role in both the discovery and the creation views regarding entrepreneurship, the task of predicting efforts toward a new venture start-up is based on the ability to perceive entrepreneurial opportunities in the market or of changes in technology or consumer needs (Edelman and Yli-Renko, 2010). In this vein, a potential entrepreneur’s efforts to create a new venture are triggered by perceptions of opportunity. The intention and energy required from potential entrepreneurs to start a new business are strengthened by stronger perceptions of opportunity (Edelman and Yli-Renko, 2010). According to Krueger et al. (2000), the intention-based cognitive process could be stimulated by the perceptions of opportunity, resulting in entrepreneurial action. Given that OR and EI are closely related (Bird, 1988), an individual who perceives a business opportunity to be desirable and feasible is more likely to create a new firm (Bhave, 1994). This reasoning indicates that university students who have a higher level of OR have stronger intentions to create a new venture.

Hence, drawing on the above results and arguments as a whole, this study proposes that individual-level OR represents a conduit through which EE realizes its impacts on EI. Hence, this study proposes the following hypothesis:

*Hypothesis* 2: Opportunity recognition mediates the cross-level relationship between entrepreneurship education and entrepreneurial intention.

### The Moderated Effect of Entrepreneurial Learning

The sustainable competitive advantage of organizations could stem from learning, considering that the extent to which it generates difficult-to-acquire but useful knowledge in individuals (Hitt et al., 2001; Hatch and Dyer, 2004). EL is defined as the process through which new knowledge is obtained by direct experience and observing the behaviors and actions of others; new knowledge is assimilated to confront discrepancies that are common when information is acquired in uncertain situations; and assimilated knowledge is organized by associating it with preexisting structures (Holcomb et al., 2009). In this sense, this study is based on two distinct theoretical frameworks for EL: experiential learning and vicarious learning.

In the case of experiential learning in an entrepreneurial setting, such learning takes place when experience and newly accumulated knowledge or memories are learned by entrepreneurs (Kolb, 1984), which can be divided into two elements: prior knowledge and the processes employed to acquire, assimilate, and organize new knowledge. From this perspective, learning requires a figurative representation of experience and then the transformation of that representation. In this sense, learning is viewed as the transformation of experiences into knowledge, and thus learning processes could consistently create new knowledge. Experiential learning offers a platform for variations in accumulated knowledge by examining the ways in which experience is converted into knowledge. Evidence supporting the experiential effects on the process of searching in the context of corporate ventures, Ronstadt (1988) noted that compared with entrepreneurs with less experience, experienced entrepreneurs could create more new businesses. Kaish and Gilad (1991) found that prior to taking action, entrepreneurs seek information, while the amount and nature of this information are influenced by the experience of entrepreneurs.

Replenishing the richness of experiential learning, entrepreneurs can also learn by observing the behaviors and actions of others (Bandura, 1977). Vicarious learning in an entrepreneurial setting occurs when the behaviors or actions of other people are the focus of attention, resulting in the information being retained, assimilated, and organized in memory. Key aspects of vicarious learning include taking an observed behavior or action as a model, assimilating and organizing these behaviors or actions, and modeling them different contexts. Research indicates that many complex behaviors can be learned by modeling others (e.g., Nadler et al., 2003), and when learning by observing others’ behaviors or actions, people gain a great deal of knowledge vicariously (Kim and Miner, 2007). Indeed, people could reduce their uncertainty by means of vicarious learning and thus by adapting this domain-specific knowledge to their own domain. Additionally, some research results show that not only observing successes but also observing both failure and near-failure could benefit observers (Kim and Miner, 2007).

According to Holcomb et al. (2009), considering that differences among people in terms of knowledge affect the opportunities that they discover and their capability to exploit these opportunities, cognitive limits and judgments as a result of biased learning might lead to the failure to recognize an opportunity. In line with this reasoning, this study emphasizes the impact of EL on whether OR is influenced positively or negatively by EE programs, which might in turn promote subsequent outcomes. This study argues that university students who are high in EL *via* experiential learning and vicarious learning might be aided in transforming knowledge or information received from EE programs into the capability to recognize business opportunities. In other words, EL is a key individual-level characteristic that could impact whether university students amplify or attenuate their capability to recognize business opportunities in the context of the influence of EE programs.

Consequently, this study suspects that university students who are high in EL are more likely to recognize business opportunities. Specifically, university students with a higher level of EL would be more sensitive to dynamic changes in situations and have more cognitive resources available to aid them in actively engaging in recognizing business opportunities. Conversely, university students who are low in EL show that they lack cognitive resources that could help them to respond favorably to surrounding situations. Accordingly, they might fail to gain access to personal resources and develop belief in the performance of OR tasks. In other words, university students who are low in EL are more likely to exhibit a passive response approach (e.g., withdrawal behavior) in that they might experience doubt about their capability to respond favorably to potential opportunities, resulting in avoidance or a decrease in OR.

Thus, drawing on the above discussions, this study proposes the following hypothesis:

*Hypothesis* 3: The relationship between entrepreneurship education and entrepreneurial intention is moderated by entrepreneurial learning, such that the relationship is more positive for university students who are high in entrepreneurial learning and more negative for university students who are low in entrepreneurial learning.

EL is considered to function as a boundary condition that influences the link between the fluctuating effectiveness of EE programs and the amplification or attenuation of engagement in OR by university students. Additionally, this study is interested in the moderating role of EL in predicting the cross-level relationships among EE programs, OR, and the EI of university students. Given that EL has already been shown theoretically to play a predictive role in facilitating OR (Politis, 2005), this perspective is advanced by arguing that university students who are high in EL are more likely to benefit from their knowledge and information in terms of effectively inspiring themselves to engage in recognizing business opportunities than are students who are low in EL. OR is assumed to be more activated by EE programs for university students who are high in EL, which subsequently motivates their intention toward entrepreneurship.

Integrating the research on EE and its influences on OR, the literature on EL, and the literature on the antecedents of EI, this study proposes a multilevel-moderated mediation model: the effect of EE programs on university students’ EI is mediated by university students’ OR, and the direction of this effect relies on university students’ EL.

*Hypothesis* 4: Entrepreneurial learning moderates the cross-level indirect effect of entrepreneurship education on entrepreneurial intention through a change in opportunity recognition. Specifically, the cross-level indirect effect is positive for university students who are high in entrepreneurial learning but negative for students who are low in entrepreneurial learning.

## Research Design and Methods

### Sample Selection

The quantitative approach was applied to obtain empirical evidence for the influences of EE on EI. Questionnaires were distributed to university students in the Guangdong-Hong Kong-Macao GBA of China to collect survey data. The survey participants had taken some type of entrepreneurship-related course or program.

The GBA is composed of the Hong Kong and Macao Special Administrative Regions and nine Pearl River Delta cities in Guangdong Province and is one of the regions with the highest degree of openness and the strongest economic vitality in China. Currently, the GBA has attracted an influx of entrepreneurs and potential entrepreneurs from around the world. As such, there are a variety of entrepreneurial activities and a strong climate toward entrepreneurship in the GBA, which may have the potential to affect the attitudes and behaviors of university students in this region.

Particularly, universities in the GBA are concerned with university-based EE, and thus, university students have ample opportunities to participate in abundant entrepreneurship-related education courses and practical programs designed to raise awareness of entrepreneurship as an alternative career and to support start-up venture projects. Thus, universities located in the GBA offer a well-matched setting for this study to explore how university-based EE influences university students.

### Procedures

To ensure sufficient representativeness and variability of survey data, 55 universities were randomly drawn from the GBA region of China, and our survey was mainly concerned with four types of student major: business management, science and technology, pharmaceutics, and literature and law. Trained interviewers conducted the survey *via* both paper-based and online-based questionnaires to collect individual-level data from university students who had generally taken some type of entrepreneurship-related lecture or program.

Considering the privacy of the survey participants, all the surveys in the study were anonymous and did not include any elements that could be used for individual identification. Furthermore, survey participants were assured of the anonymous nature of the data collection effort in advance. All survey participants were informed that participation was voluntary and that confidentiality was ensured. Per applicable institutional and national guidelines, no additional consent was required.

A total of 1,150 valid questionnaires from 1,885 respondents were collected, giving a response rate of 61%. Our final sample consisted of 736 male and 414 female students; undergraduate students accounted for 83% of participants, and postgraduate students accounted for 17%. In terms of student major, business management accounted for 45%, science and technology accounted for 23%, pharmaceutics accounted for 7%, and literature and law accounted for 25%. Among participants, 79% had no prior entrepreneurial experience, and 21% had some experience with entrepreneurship. According to Ajzen (1991), considering that respondent students generally have approximately 1 year on average until they make a decision concerning their next career, self-assessed EI was assumed to be a valid predictor of actual behavior.

### Measures

The original English scales were translated into Chinese for the survey. In accordance with Brislin (1980), bilingual experts conducted the translation–back translation procedure to confirm the accuracy of the translation. A 5-point Likert scale was used to measure model constructs, and survey participants gave their responses on a scale ranging from 1 (strongly disagree) to 5 (strongly agree).

#### Entrepreneurship Education

EE was measured on a 10-item scale adopted by Franke and Lüthje (2004). This scale was designed to assess university students’ perceived EE in terms of the atmosphere of EE, psychological quality of education and the curriculum, and activity development. A sample item is as follows: “My university has a strong cultural atmosphere of innovation and entrepreneurship.” Given that EE was considered a construct at the organizational level, individual perceived EE ratings should be aggregated to a high degree. Based on a multilevel random-intercept model analysis, the results showed that the mean *R*_wg(j)_ of EE was 0.907 (SD = 0.165), indicating that there was sufficient within-group agreement to justify group-level aggregation (LeBreton and Senter, 2007). Additionally, according to the recommendation of Geldhof et al. (2014), multilevel confirmatory factor analysis (MCFA) was applied to assess Cronbach’s alpha at the within-group and between-group levels. In accordance with the MCFA approach, to prevent conflation in reliability estimates at the within-group and between-group levels, the measurement model parameters at both levels were specifically decomposed. The results indicated that Cronbach’s alpha at the between-group level was 0.964 and Cronbach’s alpha at the within-group level was 0.886, demonstrating that the measure was reliable at both levels.

#### Opportunity Recognition

Drawing on the work of Chandler and Hanks (1994), a 4-item self-assessment scale was used to measure the ability of EE participants to identify new entrepreneurial opportunities. A sample item is as follows: “I am able to accurately identify unmet customer needs.” Notably, previous empirical studies have supported the validity and reliability of the scale (Wei et al., 2019). Moreover, Cronbach’s alpha, as based on MCFA (Geldhof et al., 2014), was 0.918 at the between-group level and 0.983 at the within-group level, demonstrating that the measure was reliable at both levels.

#### Entrepreneurial Learning

Entrepreneurial learning was measured by an 8-item scale based on the concept of entrepreneurial learning mentioned by Politis (2005) and Chandler and Lyon (2009). EE participants were confronted with statements regarding experiential learning and vicarious learning in an entrepreneurial setting, and they were required to indicate a mark closer to the statement that represented their state of learning. A sample item measuring experiential learning was as follows: “Existing experience (management experience, entrepreneurial experience, etc.) is very important for entrepreneurial decision-making.” A sample item measuring vicarious learning was as follows: “Observing the behavior of others (including failure behavior) is an important source of information.” Cronbach’s alpha, as based on MCFA (Geldhof et al., 2014), was 0.927 at the between-group level and 0.829 at the within-group level, demonstrating that the measure was reliable at both levels.

#### Entrepreneurial Intention

EI was defined as an individual’s subjective likelihood of participating in entrepreneurship-related activities within 5 years of successful completion of university study. This definition takes into account the fact that intentions are more measurable without unpredictable time lag and potential survival bias, ex post rationalization by the respondents, or the risk of identifying the consequences instead of the determinants of self-employment. Therefore, intentions are likely to directly reflect the influences of organizational-level factors (e.g., EE) (Walter et al., 2013); thus, this study focused on EI as an outcome variable. EI was measured on a 4-item scale developed by Kuckertz and Wagner (2010). A sample item is as follows: “I am determined to create a firm in the future.” Furthermore, Cronbach’s alpha, based on MCFA (Geldhof et al., 2014), was 0.921 at the between-group level and 0.886 at the within-group level, demonstrating that the measure was reliable at both levels.

#### Control Variables

Based on recent studies concerning the relationship between gender and entrepreneurship, entrepreneurship is considered to be a masculine field (Gupta et al., 2009; Hu et al., 2018; Wu et al., 2019). According to Unger et al. (2011), investments in human capital can enhance the benefits of entrepreneurship, and thus, EE participants’ education levels (grades) and majors should be considered. Additionally, intention to engage in entrepreneurial activities may be more likely to be influenced by respondents’ entrepreneurial experience or work experience (Kautonen et al., 2015). Furthermore, following the recommendation of Walter et al. (2013), respondents’ need for achievement and entrepreneurial role models are considered to be important individual-level influences. Taken together, this study measured and controlled for EE participants’ gender, education level, major, entrepreneurial experience, need for achievement, and entrepreneurial role models.

### Analytical Strategy

Based on nested data (e.g., students nested within universities), this study attempted to explore the impact of organizational-level EE on individual-level EI *via* a multilevel approach. HLM has been recommended to deal with nested data and to conduct cross-level analysis (Hofmann, 1997; Raudenbush and Bryk, 2002). In addition, intercepts-as-outcomes models were employed to test our hypotheses in light of the fact that the main goals of the study were to evaluate the influences of organizational-level predictors on individual-level outcomes.

Unlike prior quasi-experimental studies, in the multilevel study design, organizational-level influences are linked to between-organization variances, and thus, significant links could be attributed to organizational factors. In this sense, the multilevel approach could further promote the establishment of the external validity of the research findings by drawing on multiorganizational samples and controlling for individual-level influences.

According to the recommendation of Enders and Tofighi (2007), grand mean centering was applied to analyze the nested data to assess the cross-level direct effects and indirect effects of predictors. Furthermore, following the recommendations of Preacher et al. (2010), bias-corrected Monte Carlo parametric bootstrapping with 20,000 resamples was applied to create a confidence interval (CI) of 95% for each simulated indirect effect to check cross-level mediation hypotheses.

## Results

### Convergent and Discriminant Validity

Following the procedure of structural equation model analysis, criteria of reliability, convergent validity, and discriminant validity were gaged to guarantee the adequacy of the construct measurement models. According to Fornell and Larcker (1981), Cronbach’s alphas and composite reliability coefficients of model constructs met the recommended criteria, indicating acceptable internal consistency. All the factor loadings of the model constructs were beyond the threshold value, and the square multiple correlation (SMC) values were over the recommended value, demonstrating acceptable item reliability. Moreover, the average variance extracted (AVE) of the model constructs were all beyond the threshold value, confirming convergent validity. Table 1 shows the overall reliability of the constructs and the factor loadings of indicators.

**Table 1:** Overall reliability of the constructs and factor loadings of indicators.

Construct (source)	Items	Factor loading	SMCs	Cronbach’ alpha	CR	AVE
Entrepreneurship education Franke and Lüthje (2004)	EE1	0.889	0.790	0.958	0.964	0.727
	EE2	0.879	0.773			
	EE3	0.875	0.766			
	EE4	0.864	0.746			
	EE5	0.859	0.738			
	EE6	0.855	0.731			
	EE7	0.827	0.684			
	EE8	0.826	0.682			
	EE9	0.826	0.682			
	EE10	0.821	0.674			
Opportunity recognition Chandler and Hanks (1994)	OI1	0.917	0.841	0.921	0.944	0.809
	OI 2	0.916	0.839			
	OI 3	0.884	0.781			
	OI 4	0.880	0.774			
Entrepreneurial learning Politis (2005) and Chandler and Lyon (2009)	EL1-1	0.845	0.714	0.920	0.953	0.717
	EL1-2	0.839	0.704			
	EL1-3	0.837	0.701			
	EL1-4	0.827	0.684			
	EL2-1	0.882	0.778			
	EL2-2	0.878	0.771			
	EL2-3	0.852	0.726			
	EL2-4	0.812	0.659			
Entrepreneurial intention Kuckertz and Wagner (2010)	EI1	0.898	0.806	0.889	0.904	0.777
	EI2	0.896	0.803			
	EI3	0.892	0.796			
	EI4	0.838	0.702			

SMCs, Square multiple correlations, CR, Composite reliability, and AVE, Average variance extracted.

Furthermore, confirmatory factor analysis was conducted to evaluate the fit of the four-factor model. The four-factor model, including EE, OI, EL, and EI, showed acceptable model fit (*χ*^2^ (98) = 180.33, GFI = 0.972, TLI = 0.966, RMSEA = 0.050, SRMR = 0.038), indicating convergent validity. To examine the discriminant validity, the four-factor model was compared with alternative models, including 43-factor models and 22-factor and 11-factor models. The first two-factor model was obtained by combining OR, EL, and EI into one latent factor, and the second two-factor model was obtained by combining EE, OR, and EL into one latent factor in terms of correlation among model constructs. In a similar vein, 43-factor models were obtained. Finally, the one-factor model was obtained by combining all the items of the model constructs into one single factor (see Table 2).

**Table 2:** Results of confirmatory factor analysis.

CFA model	*χ* ^2^	df	CFI	TLI	RMSEA	SRMR
One-factor model	1616.76	104	0.612	0.552	0.210	0.120
EE, OR, EL, and EI were blended						
Two factor model	1149.75	103	0.731	0.687	0.175	0.096
OR, EL, and EI were blended						
Two factor model	1199.02	103	0.719	0.672	0.180	0.100
EE, OR, and EL were blended						
Three-factor model	747.56	101	0.834	0.803	0.139	0.076
OR and EL were blended						
Three-factor model	669.25	101	0.854	0.827	0.131	0.070
OR and EI were blended						
Three-factor model	805.91	101	0.819	0.785	0.145	0.091
EL and EI were blended						
Three-factor model	751.03	101	0.833	0.802	0.140	0.083
EE and EL were blended						
Four-factor model	180.33	98	0.972	0.966	0.050	0.038
Four-factor model + Method factor	167.24	90	0.974	0.965	0.051	0.036

*χ*^2^, Chi-square value; df, Degree of freedom; CFI, Confirmatory fit indices; TLI, Tucker-Lewis index; RMSEA, Root mean square error of approximation; and SRMR, Standardized root mean square residual. EE, Entrepreneurship education; OR, Opportunity recognition; EL, Entrepreneurial learning; and EI, Entrepreneurial intention.

Additionally, to check the common method bias, according to the recommendation of Podsakoff et al. (2003), the study controlled for the effects of a single unmeasured latent method factor by adding a first-order factor with all of the constructs’ measures as indicators to the proposed theoretical model. This technique is a latent variable approach that has been widely applied by a few empirical studies (e.g., Carlson and Kacmar, 2000; Conger et al., 2000). The results showed that the model (the four-factor model added the method factor) did not have a stronger model fit than the proposed four-factor model, demonstrating that common method bias was not an issue (see Table 2).

Table 3 presents descriptive statistics and intercorrelations among all variables. The results showed that EE was positively related to OR (*r* = 0.520, *p* < 0.01) and EI (*r* = 0.360, *p* < 0.01). EL was positively related to OR (*r* = 0.627, *p* < 0.01) and EI (*r* = 0.507, *p* < 0.01). Furthermore, OR was positively related to EI (*r* = 0.590, *p* < 0.01).

**Table 3:** Correlations matrix of main variables and discriminant validity by Fornell–Larcker criterion.

S. no	Construct	Mean	SD	1	2	3	4	5	6	7	8	9	10
1.	Gender	1.38	0.45	1									
2.	Education	2.36	1.31	−0.020	1								
3.	Major	2.58	1.16	0.318^**^	0.023	1							
4.	EN-Ex	1.25	0.42	0.166^**^	0.078	0.019	1						
5.	NA	3.30	0.78	0.065	−0.021	−0.023	0.187^**^	1					
6.	RMP	2.55	0.81	0.122^*^	−0.035	0.037	0.195^**^	0.102^*^	1				
7.	EE	3.46	0.76	0.017	−0.064	0.113^*^	0.150^**^	0.009	0.035	**0.853**			
8.	OR	3.34	0.78	0.096	0.055	0.057	0.190^**^	0.178^**^	0.115^**^	0.520^**^	**0.899**		
9.	EL	3.45	0.73	0.085	−0.053	0.035	0.200^**^	0.105^**^	0.106^*^	0.490^**^	0.627^**^	**0.847**	
10.	EI	3.25	0.86	0.175^**^	0.022	0.078	0.197^**^	0.109^**^	0.129^*^	0.360^**^	0.590^**^	0.507^**^	**0.881**

The square roots of AVE (the bold numbers) for discriminant validity are along the diagonal. Gender: 1 = female (36%), 2 = male (64%). Education: 1 = grade 1 (4%), 2 = grade 2 (15%), 3 = grade 3 (42%), 4 = grade 4 (22%), and 5 = post graduate (17%). Major: 1 = literature and law (25%), 2 = pharmaceutics (7%), 3 = business management (45%), and 4 = science and technology (23%). Entrepreneurial experience (EN-Ex): 1 = none (79%), 2 = have (21%). NA, need for achievement; RMP, role model performance. EE, Entrepreneurship education; OR, Opportunity recognition; EL, Entrepreneurial learning; and EI, Entrepreneurial intention.

* *p* < 0.05;

** *p* < 0.01.

In addition, following the recommendation of Fornell and Larcker (1981), to further evaluate discriminant validity, the square roots of AVE were compared with interconstruct correlations. The results showed that the square root of the AVE of a construct was beyond all cases of interconstruct correlation coefficients (see Table 3). As such, all model constructs were presumed to reach acceptable discriminant validity.

### Hypothesis Testing

Due to the analysis of cross-level data, HLM was considered to make it more appropriate for multilevel analysis to test the hypotheses (Hox, 2002). First, to examine the presence of between-group variance of model variables and to check the significance of between-level residual variance, a null model analysis was conducted to examine the intraclass correlation coefficients (ICC1). The results of the null model analysis showed that the ICC1 values of OR, EL, and EI are 0.37, 0.31, and 0.35, respectively, indicating that there is enough variance to be explained at between-group and within-group levels to support using multilevel analysis (Bryk and Raudenbush, 1992). The [intraclass correlation (2)] values of OR, EL, and EI were 0.83, 0.65, and 0.78, respectively, indicating acceptable reliability of the group-level constructs (James et al., 1992).

Hypothesis 1 claimed that organizational-level EE is positively associated with individual-level EI. As shown in Model 3 in Table 4, the results showed that the relationship between EE and EI was not significant when controlling for individual-level OR (*β* = 0.165, SE = 0.099, *p* > 0.05). In addition, 24.6% of the variance in EI at the between-group level was explained by predictors in Model 3. This result indicated that university students with a high level of EE are not more likely to have a high level of EI. Thus, Hypothesis 1 was not confirmed.

**Table 4:** Results of multilevel regression analysis with entrepreneurship education as independent variable.

	Mediator: Opportunity recognition						DV: Entrepreneurial intention		
	Model1			Model 2			Model 3		
	*B*	SE	*t*	*B*	SE	*t*	*B*	SE	*t*
**Main effect within level**									
Gender	0.022	0.069	0.322	0.010	0.070	0.148	0.200	0.086	2.314^*^
Education	0.042	0.024	1.734	0.043	0.024	1.810	0.021	0.030	0.694
Major	0.035	0.028	1.219	0.034	0.028	1.200	0.037	0.035	1.048
EN-Ex	0.083	0.080	1.036	0.073	0.080	0.907	0.139	0.100	1.397
NA	0.114	0.059	1.932	0.150	0.081	1.851	0.233	0.074	3.148^**^
RMP	0.145	0.058	2.500^*^	0.183	0.071	2.569^*^	0.084	0.073	1.155
OR							0.650	0.052	12.474^***^
EL				0.652	0.046	14.164^***^			
**Main effect between level**									
EE	0.461	0.102	4.530^***^	0.274	0.081	3.372^**^	0.165	0.099	1.664
**Cross-level moderation**									
EE × EL				0.226	0.105	2.148^*^			
Within group R^2^	0.024			0.395			0.358		
Between group R^2^	0.589			0.684			0.246		

Calculations are based on *N* = 55 at the organizational level and *N* = 1,150 at the individual level. EN-Ex, Entrepreneurial experience; NA, Need for achievement; RMP, Role model performance; EE, Entrepreneurship education; OR, Opportunity recognition; EL, Entrepreneurial learning; and EI, Entrepreneurial intention.

* *p* < 0.05;

** *p* < 0.01;

*** *p* < 0.001.

Hypothesis 2 proposed that OR would mediate the cross-level relationship between EE and EI. Following the recommendation of MacKinnon et al. (2002), the product of coefficients of the path EE → OR and the path OR→EI was calculated to check the indirect effect. The results showed that the indirect effect was significant for the relationship between EE and EI (indirect effect = 0.300, *p* < 0.001). In addition, based on bias-corrected Monte Carlo parametric bootstrapping with 20,000 resamples, the 95% confidence interval (CI) excluded zero (95% CI = [0.166, 0.458]). Thus, the results supported the inference of indirect effects. Furthermore, aside from the significant indirect effect *via* OR, EE did not have a significant direct effect on EI (*β* = 0.165, SE = 0.099, *p* > 0.05), indicating full mediation. Thus, Hypothesis 2 was supported.

**Figure 2:**
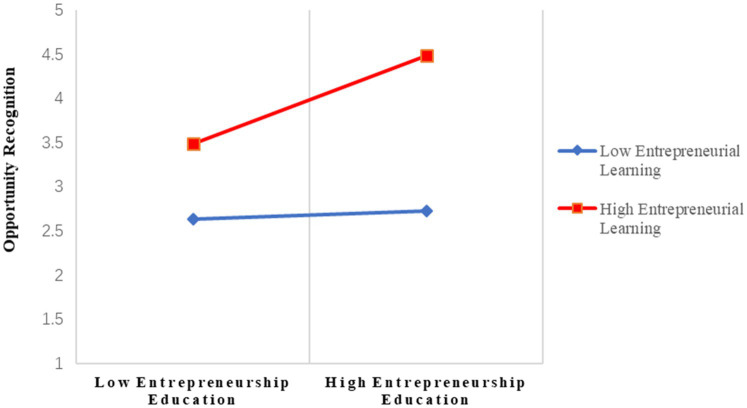
Entrepreneurial learning as a moderator of the cross-level relationship between entrepreneurship education and opportunity recognition. Entrepreneurial learning was centered at groups’ mean values. Entrepreneurship education was centered on the mean of the whole sample of universities.

Hypothesis 3 proposed that EL moderates the cross-level relationship between EE and OR. The results of the cross-level moderation analysis are presented in Table 3. The cross-level relationship was predicted to be significantly positive for EE participants with a high level of EL and nonsignificantly positive for EE participants exhibiting a low level of EL. As shown in Model 2 in Table 4, EL acted as a moderator of the cross-level relationship between EE and OR (*β* = 0.226, SE = 0.105, *p* < 0.05). In addition, 68.4% of the variance in OR at the between-group level was explained by predictors in Model 2. The results indicated that for EE participants with a high level of EL, the relationship between EE and OR was positive, but for EE participants showing a low level of EL, the relationship was not significant. Thus, Hypotheses 3 was supported. Following Aiken and West’s (1991) procedure, the moderation effect was plotted by computing slopes one standard deviation above and below the mean of the moderator (EL). The cross-level moderation effect is presented in Figure 2.

Hypothesis 4 predicted that EL moderates the cross-level indirect effect of EE on EI through changing levels of OR, which was the hypothesis of conditional indirect effects. To test this hypothesis, the indirect effects of EE on EI *via* OR were calculated in terms of both the high value of the moderator (mean of EL + 1 SD) and the low value of the moderator (mean of EL − 1 SD). As shown in Table 5, the results show that the indirect effect was significantly positive for EE participants high in EL (indirect effect: *b* = 0.286, SE = 0.084, *p* < 0.01). In addition, based on bias-corrected Monte Carlo parametric bootstrapping with 20,000 resamples, the 95% confidence interval (CI) excluded zero (95% CI = [0.133, 0.464]). In contrast, the indirect effect was nonsignificant for EE participants low in EL (indirect effect: *b* = 0.071, SE = 0.080, *p* > 0.05). In addition, the 95% bias-corrected Monte Carlo parametric bootstrap confidence interval (CI) was [−0.080, 0.233], which included zero. This result indicated that the indirect effect is much stronger when EE participants exhibit a high level of EL than when EE participants exhibit a low level of EL. According to the recommendation of Hayes (2015), the index of moderated mediation was calculated to further test the effect of moderated mediation. The results showed that the index of moderated mediation was 0.147 (SE = 0.069), and the 95% bias-corrected Monte Carlo parametric bootstrap confidence interval (CI) was [0.013, 0.286], which included zero. Based on this evidence, it could be concluded that the indirect effect of EE on EI *via* OR is strongly moderated by EE participants’ EL. Thus, Hypotheses 4 was supported.

**Table 5:** Conditional indirect effects of entrepreneurship education on entrepreneurial intention through opportunity recognition for different values of entrepreneurial learning.

Moderator: Entrepreneurial learning			
	Unstnd. Estimate(*b*)	SE	95% CI for Mean Estimate^a^
−1SD	0.071	0.080	[−0.080, 0.233]
Mean	0.178	0.057	[0.078, 0.301^*^]
+1SD	0.286	0.084	[0.133, 0.464^*^]

Calculations are based on *N* = 55 at the organizational level and *N* = 1,150 at the individual level. Significant indirect effects using Monte Carlo confidence intervals. Unstnd, Unstandardized. EE, Entrepreneurship education; OR, Opportunity recognition; EL, Entrepreneurial learning; and EI, Entrepreneurial intention.

a There values are based on the unstandardized path coefficients.

* 95% confidence intervals exclude zero.

## Discussion

How do university students respond to EE programs in terms of OR and EI? Furthermore, does this response change based on their level of EL? The effectiveness of EE programs is a significant topic in the domain of entrepreneurship research, yet current theory and empirical research have provided mixed results and evidence regarding the factors and underlying mechanisms explaining when and how EE programs lead to enhanced OR and subsequently foster EI. This study attempts to offer competing theoretical explanations and expand knowledge on the cross-level links between EE programs and university students’ EI. By proposing and testing a multilevel-moderated mediation model, this study specifies how EE programs influence university students’ EI and how EL facilitates OR and EI in university students.

### Research Implications

Drawing on multiple studies, this study attempts to evaluate the effectiveness of EE programs on the EI of university students. Specifically, our multilevel-moderated mediation model is proposed by incorporating the perception of OR and considering the moderating role of EL. To address this research goal, HLM is employed to test our research predictions on the basis of a survey of 1,150 university students from 55 universities in the GBA of China. The findings show that EE programs can enhance the EI of university students *via* OR. Furthermore, EL plays a moderating function in the cross-level relationship between EE and OR.

This study makes several crucial contributions to the EE literature. First and foremost, the findings of this study offer empirical evidence concerning ongoing disputes regarding the teachability of entrepreneurship (Aronsson, 2004; Gendron, 2004; Henry et al., 2005). When considering the premise that the EI of university students could be enhanced by organizational-level factors while controlling for individual-level influences, drawing on the human capital, OR, EL, and organizational norms literature, this study proposes a multilevel model to examine how EE as a between-group influence fosters the within-group intentions of university students. Based on the nested survey dataset (students nested within universities), a multilevel model approach is applied to provide empirical evidence to support the effectiveness of EE programs in fostering EI among university students. Hence, this study complements previous research, which has mostly taken the form of ex ante and ex post-surveys (e.g., Kolvereid and Moen, 1997; Menzies and Paradi, 2003) and case-based evidence (e.g., Peterman and Kennedy, 2003) by means of large-scale surveying of a representative, multiuniversity, and cross-level sample to warrant the generalizability of findings and the richness of the EE literature to support a conventional conjecture regarding the effectiveness of EE.

Second, this study offers further empirical evidence to support the claim that, like entrepreneurial capability, OR can be learned and fostered (e.g., DeTienne and Chandler, 2004; Morris et al., 2013). OR could stimulate the entrepreneurial process (Ramos-Rodríguez et al., 2011), and the combination of individuals and opportunities could generate entrepreneurship (Shane and Venkataraman, 2000; Shane, 2012). Hence, developing the identification and evaluation of opportunities is essential for EE programs. However, there is still insufficient understanding of how EE programs influence OR and the mediating role of OR in the cross-level relationship between EE programs and EI.

According to the EE literature and research (Politis, 2005; Krueger, 2007), EE programs, in the form of case studies, interacting with entrepreneurs, and activities regarding entrepreneurship, could offer field-specific experiences that lead to the development of entrepreneurial awareness. Indeed, entrepreneurial awareness could be enhanced by emphasizing cognitive mechanisms and promoting OR (Baron, 2006), which is considered to be the first stage of the entrepreneurial process and a key stimulator of entrepreneurial activities (Costa et al., 2018). Cognitive frameworks have been considered crucial for facilitating entrepreneurial awareness, which could orient individuals to be sensitive or alert to unique or specific information in unique contexts, resulting in OR (Baron, 2006). In this sense, EE programs or training could influence participants’ development of cognitive mechanisms, which would result in fostering their OR.

In line with this reasoning, by introducing the perception of OR as a proximal cause of EI, this study explores the indirect effectiveness of EE programs on EI *via* OR. Our findings further provide supporting empirical evidence to aid in exploring and uncovering the underlying mechanism by which EE programs, as a contextual influence, impact the development of EI. Drawing on the above arguments, the findings of our study also contribute to the OR literature.

Third, this study examines EL as a boundary condition in describing why university students respond differently to the influences of EE programs in terms of OR and EI, thus extending the EL literature.

EL not only provides a foundation to examine the variations in accumulated knowledge whereby experience is transformed into knowledge (e.g., experiential learning; Holcomb et al., 2009) but also helps people access a great deal of knowledge by observing others (e.g., vicarious learning; Kim and Miner, 2007; Holcomb et al., 2009) and to adapt this domain-specific knowledge to their own field as a means of reducing their uncertainty. Given that individual differences in knowledge influence individuals’ capability to discover and exploit opportunities (Holcomb et al., 2009), EL could expand their cognitive limits and enhance their judgments, resulting in effective recognition of business opportunities.

Nonetheless, prior studies have indicated that EL plays a significant role in predicting OR (Politis, 2005), and there is only a small body of research concerning the moderated function triggered by university students high versus low in EL when faced with EE programs that are perceived to be more or less intense. This study argues that university students’ differences in terms of EL moderate the influencing process of EE programs, such that the links between intensity in perceived EE, OR, and EI were stronger for those with high EL versus those with low EL.

In line with this reasoning, our findings show that EL substantially benefits university students’ capability to recognize business opportunities when they are influenced by a higher intensity of EE programs than usual or at a relatively low level. The higher intensity of EE programs might facilitate participants’ activation, alertness, and perseverance for university students who are high in EL, resulting their being able to work harder toward recognizing business opportunities. As such, individuals who are high in EL may take OR as a functional approach to increase their intention toward entrepreneurship. By further exploring the interplay between EE and EL, our findings indicate that university students’ EL is not an equally important resource in all kinds of EE contexts. Hence, introducing EL as a boundary condition at the individual level could further benefit our understanding of how interindividual differences in this personal resource affect the link between EE and EI.

Based on the EE and psychology literature, this study advances extant research into the mechanism underlying how university students respond to perceived EE programs by examining OR as a mediating role. Furthermore, this study extends prior research on the role of EL by investigating its function as a boundary condition that could alter the effects of perceived EE programs on levels of OR and EI. Our findings further aid in better understanding the process by which university students’ EI develops.

### Practice Implications

In addressing our research question—How do EE programs inspire prospective entrepreneurs?—our research findings have critical practical implications for policy-makers who must make decisions concerning the allocation of scarce education resources and who are dedicated to encouraging students toward entrepreneurship. By adopting educational activities and appropriate teaching approaches, EE programs could play a substantial role in fostering university students’ EI. Our results strongly indicate that taking part in entrepreneurial training programs could significantly enhance university students’ OR and EI, confirming that entrepreneurship schooling can foster and shape potential entrepreneurs.

According to Shane and Venkataraman (2000), OR has been considered the core of entrepreneurship; however, there is only a small body of research that emphasizes training university students in how to identify or create new business opportunities (Neck and Greene, 2011). Considering the impacts of EE programs on OR, EE educators or trainers should pay more attention to enhancing this capability in the design and implementation of their programs. Nonetheless, a recent research survey showed that competency in terms of OR has often been omitted or received less attention during EE programs or courses (Karimi et al., 2016). According to Neck and Greene (2011), the most common EE programs emphasize opportunity exploitation on the basis of the assumption that business opportunities have already been recognized. In this case, creativity, the process of idea generation, and entrepreneurial opportunity recognition have received little attention.

Hence, the task of fostering OR competence should be introduced into the design principle of EE programs and identified as an especially key component of EE. Additionally, following the recommendation of Karimi et al. (2016), OR competence could be improved by teaching creativity skills. A portfolio of classroom teaching activities, such as creativity-related activities (e.g., divergent thinking and idea generation exercises), and field experiential activities, such as internships in new ventures, engagement with community entrepreneurs, and guest lectures and mentoring by local entrepreneurs, have been considered effective means that could enhance university students’ competence in recognizing business opportunities (Sardeshmukh and Smith-Nelson, 2011). Moreover, the role of networking has been regarded as another factor in enhancing OR. According to Ozgen and Baron (2007), research has shown that social networking plays a substantial part in the process of recognizing business opportunities. Social networking has been closely related to the performance of opportunity identification (Ozgen and Baron, 2007; Karimi et al., 2016) because both knowledge and new ideas can originate from social networks (Johannisson, 1990).

As such, entrepreneurship educators should introduce the task of fostering networking skills in university students into the design of EE programs and offer more chances and more opportunity to network with entrepreneurship-friendly peers and entrepreneurs.

Our findings also highlight the benefits of enhancing university students’ EL, which leads to improved OR and EI. According to the EL literature, EL is considered a moderately tenacious individual characteristic that could be improved by specific interventions in the form of direct learning experiences. As such, entrepreneurship educational interventions should attempt to enhance university students’ entrepreneurship-related knowledge, abilities, and skills aimed at improving their entrepreneurial competencies. In this regard, entrepreneurship education programs should offer “real-world” “virtual” experiences in the classroom in the forms of roleplaying, case studies, and real business simulations (Karimi et al., 2016) to enhance and strengthen entrepreneurial experience learning through mastery experiences or repeated performance accomplishments. According to Gielnik et al. (2015), it is recommended to implement an entrepreneurial training intervention to offer real experience in starting a real venture to increase students’ EL (e.g., experience learning). University students could be enabled to directly learn and receive entrepreneurship-related knowledge in the process of starting and running a real business by engaging in a structured entrepreneurial program that offers support and guidance.

Furthermore, the EL literature indicates that EL could be enhanced when potential entrepreneurs observe and imitate role models that are skilled at dealing with complex situations associated with running a real business. Hence, observing, listening to, and being mentored by those role models might aid potential entrepreneurs in fostering EL (e.g., vicarious learning). In this sense, entrepreneurial training programs should provide opportunities for university students to interact with real entrepreneurs, to engage in networking with entrepreneurially minded friends and peers, and to interact with entrepreneurs as role models (Souitaris et al., 2007; Mueller, 2011; Karimi et al., 2016), and entrepreneurship educational activities, including real entrepreneurs as guest speakers, video profile presentations of well-known entrepreneurs, and student internships in ventures should be provided (Karimi et al., 2016). These learning approaches should be carried out in the context of entrepreneurship schooling and training programs for potential entrepreneurs, such as university students, in the context of universities and entrepreneurial ecosystem accelerators.

### Limitations and Future Directions

This study faces certain limitations that should be addressed in further research. First, given the fact that this study only evaluated the impact of participating in EE programs on university students’ OR and EI, future research opportunities should be provided for exploring specific teaching approaches, particularly design elements and the training contents of EE programs, as well as the links between these factors and student outcomes. The question of how different types of EE programs influence university students’ OR and attitudes toward entrepreneurship may be addressed by future entrepreneurship researchers.

Second, the effect of the diffusion of EE programs within universities might be omitted due to the limitations of our survey data. This study proposes that a portfolio of EE programs (e.g., teaching courses, entrepreneurs’ lectures, and field practices) could account for a major share of the between-university variance in terms of student-level program-derived benefits (e.g., OR and EI). As such, this study is concerned with program participants in one or more types of EE programs. Nonetheless, program participants are more likely to share their feelings, emotions, and views with their fellow students, resulting in the diffusion of entrepreneurship-related knowledge, attitudes, perceptions, and motivation within universities. Future searchers might explore the “contagion effect” of EE programs, that is, how EE programs inspire the diffusion of entrepreneurship-related competencies and passion within universities. In this sense, by expanding the sample survey, our findings could be further verified and extended by expanding the sample survey and comparison analysis.

Finally, considering that EI regarded as an academic field has obtained traction in the entrepreneurial behavior literature (Bird, 1988; Pham et al., 2021), however, the entrepreneurship literature has raised issues of concern associating with the effectiveness of EI in driving action (Nabi et al., 2018). Future research should expand our study by further examining the links between intention and behavior in the context of entrepreneurship and the ways in which this occurs because there is only a small body of empirical research that focuses on the process of how EI translates into entrepreneurial behavior (Nabi et al., 2017). Accordingly, future researchers are recommended to apply a longitudinal research approach to explore and explain the dynamic process of entrepreneurial attitudes and intention over time and the subsequent formation and development of entrepreneurial behavior.

## Conclusion

This study attempts to address the competing theoretical views regarding the links between EE and EI. By developing and empirically testing a multilevel-moderated mediation model, this study investigates university students’ OR as a mediating mechanism that could be activated under the influence of EE programs by university students high in EL. Our findings indicate that the extent to which EE programs enhance university students’ capability to recognize business opportunities and in turn facilitate the development of their intention toward entrepreneurship is moderated by individual characteristics of EL. This study contributes to prior research in the field of EE through a multilevel approach on the basis of a nested dataset, by examining within-group level variation in university students’ OR and EI and by introducing EL as a key boundary condition at the individual level.

## Data Availability Statement

The raw data supporting the conclusions of this article will be made available by the authors, without undue reservation.

## Ethics Statement

An ethics approval was not required as per applicable institutional and national guidelines and regulations. The informed consent of the participants was implied through survey completion.

## Author Contributions

FH take charge of the research design, methodology, literature review, analysis, and interpretation of data, as well as drafting and revising the manuscript. YS contributed to the literature review, research design, and practice implication. MQ conceived the literature review, research design, data collection, and practice implication. JC and JT conceived the literature review and data collection. All authors contributed to the article and approved the submitted version.

## Funding

This study was financially supported by innovation project of universities in Department of Education of Guangdong Province of China (grant no: 2020WTSCX121).

## Conflict of Interest

The authors declare that the research was conducted in the absence of any commercial or financial relationships that could be construed as a potential conflict of interest.

## Publisher’s Note

All claims expressed in this article are solely those of the authors and do not necessarily represent those of their affiliated organizations, or those of the publisher, the editors and the reviewers. Any product that may be evaluated in this article, or claim that may be made by its manufacturer, is not guaranteed or endorsed by the publisher.
